# The distribution pattern of periprostatic neurovascular bundles examined with successive celloidin slices

**DOI:** 10.1186/s12894-020-00778-0

**Published:** 2021-01-06

**Authors:** Xuemei Li, Jianhui Wu, Qiliang Cai, Janming Pan, Qingguo Meng, Ping Zhang, Yong Xu, Lidong Zhai

**Affiliations:** 1 NHC Key Laboratory of Hormones and Development, Tianjin Key Laboratory of Metabolic Diseases, Chu Hsien-I Memorial Hospital & Tianjin Institute of Endocrinology, Tianjin Medical University, Tianjin, 300134 China; 2 Department of Urology, Tianjin First Central Hospital, Tianjin, 300192 China; 3 Department of Urology, the Second Hospital of Tianjin Medical University, Tianjin Institute of Urology, Tianjin, 300211 China; 4 Department of Anatomy, School of Integrative Medicine, Tianjin University of Traditional Chinese Medicine, Tianjin, 301617 China; 5 Department of Anatomy and Histology, School of Basic Medical Sciences, Tianjin Medical University, 22 Qixiangtai Road, Heping District, Tianjin, 300070 China

**Keywords:** Prostatic capsule, Urinary incontinence, Prostatectomy, Neurovascular bundles, Male urethral sphincter, Detrusor apron

## Abstract

**Background:**

Although several distribution patterns of periprostatic neurovascular bundles have been proposed, variant dissection technique based on these patterns still confused surgeons. The aim of this study was to describe the periprostatic neurovascular bundles and their relationship with the fascicles around prostate and provide the accurate morphologic knowledge of periprostatic tissue for prostate operation.

**Methods:**

The pelvic viscera were obtained from 26 adult male cadavers. They were embedded in celloidin and cut into successive slices. The slices were explored with anatomic microscopy. 3-Dimensional reconstruction was achieved with celloidin sections and series software.

**Results:**

The prostatic capsule which surrounded the dorsal, bilateral aspect of the prostate was attached ventrally to anterior fibrous muscular stroma (AFMS). The lower part of the striated sphincter completely embraced the urethral; the upper part of this muscle covered the lower ventral surface of prostate. The upper ventral surface of prostate is covered by the circular muscle of detrusor. The levator fascia and the capsule adhered on the most convex region of the lateral prostate, but separated on the other region. The pelvic neurovascular bundles (PNVB) divided into the anterior and posterior divisions. The anterior division continued as dorsal vascular complex (DVC). The distal part of DVC entered into penile hilum. The posterior division continued as neurovascular bundles, and then as the cavernous supply (CS). The distal part of CS joined into pudendal neurovascular bundles.

**Conclusions:**

The capsule and AFMS formed a pocket like complex. There were anterior and posterior neurovascular approaches from PNVB to penile hilum.

## Background

The fascial fascicles around prostate, the distribution pattern of the periprostatic nerves and vessels, and adjacent relationship between them are crucial for determining the dissection technique in prostatectomy. However, the anatomy of these periprostatic structures and their relationships remains controversial, and different dissection technique proposed by several groups based on variant morphological studies confused surgeons. Walsh and Donker, who introduced nerve-sparing radical prostatectomy procedure, demonstrated two fascia layers on lateral side of prostate: the outer levator fascia and the inner prostatic fascia. Cavernous nerve situated posterolaterally to the prostate between these two layers [1]. Differently, Walz et al. illustrated three layers of distinct membranous structures. The neurovascular structures sandwiched between the prostatic fascia and levator fascia. Accordingly, they proposed intra-, inter- and extra- fascial dissection planes [2]. Menon et al. revealed a multilayer lateral prostatic fascia containing NVBs, and proposed the nerve-sparing approach “Veil of Aphrodite” [3]. Lunacek et al. showed dispersion of cavernous nerves along the prostatic capsule and recommended a “curtain dissection” [4]. Recently, five nerve-sparing grades have been reported in nerve-sparing prostatectomy based on a land mark artery and fascial structures around prostate, and four nerve-sparing grades have also been proposed according to venous system and fascial layers [5, 6].

When performing pelvic or genital surgery, knowledge of the anatomic relationship of the cavernous nerves to the membranous urethra and hilum of the penis is important [7]. The vascular and neural damage distal to prostate are responsible for the loss of penile erection in pelvic fracture urethral injury [8]. But the precise anatomy of the distal neurovascular bundles from lateral prostate and their relation to the striate sphincter, the levator ani muscle and the pudendal neurovascular bundles were still unclear.

In the present study, we used successive and multi-axis celloidin sections to observe the periprostatic tissue in overall and multi-angle view. The purpose of the research is to provide the accurate morphologic knowledge of periprostatic tissue for prostate operation.

## Methods

The pelvic organs were obtained from 26 adult male cadavers, 50–85 yr of age (mean: 66.1 yr). The cadavers were donated to Tianjin Medical University for research and education in accordance with their consent, and their use in research was approved by the Ethics Committee of Tianjin Medical University. The written informed consent was obtained from all the participants in their lifetime.

The entire intrapelvic organs were embedded in celloidin. The embedded blocks were cut into successive slices by an immersing-alcohol microtome (L-type; R. Jung AG, Heidelberg, Germany). The detailed procedures have been described in our previous articles [9, 10]. Slices were examined with microscopy (SZX7; Olympus, Tokyo, Japan) and were read by 2 blinded readers.

Pelvic structures were outlined manually for all sections and reconstructed in 3D using Mimics 19.0 software (Materialise Inc., Belgium). The complete 3D reconstruction was performed in three adult specimens.

## Results

**Fig. 1:**
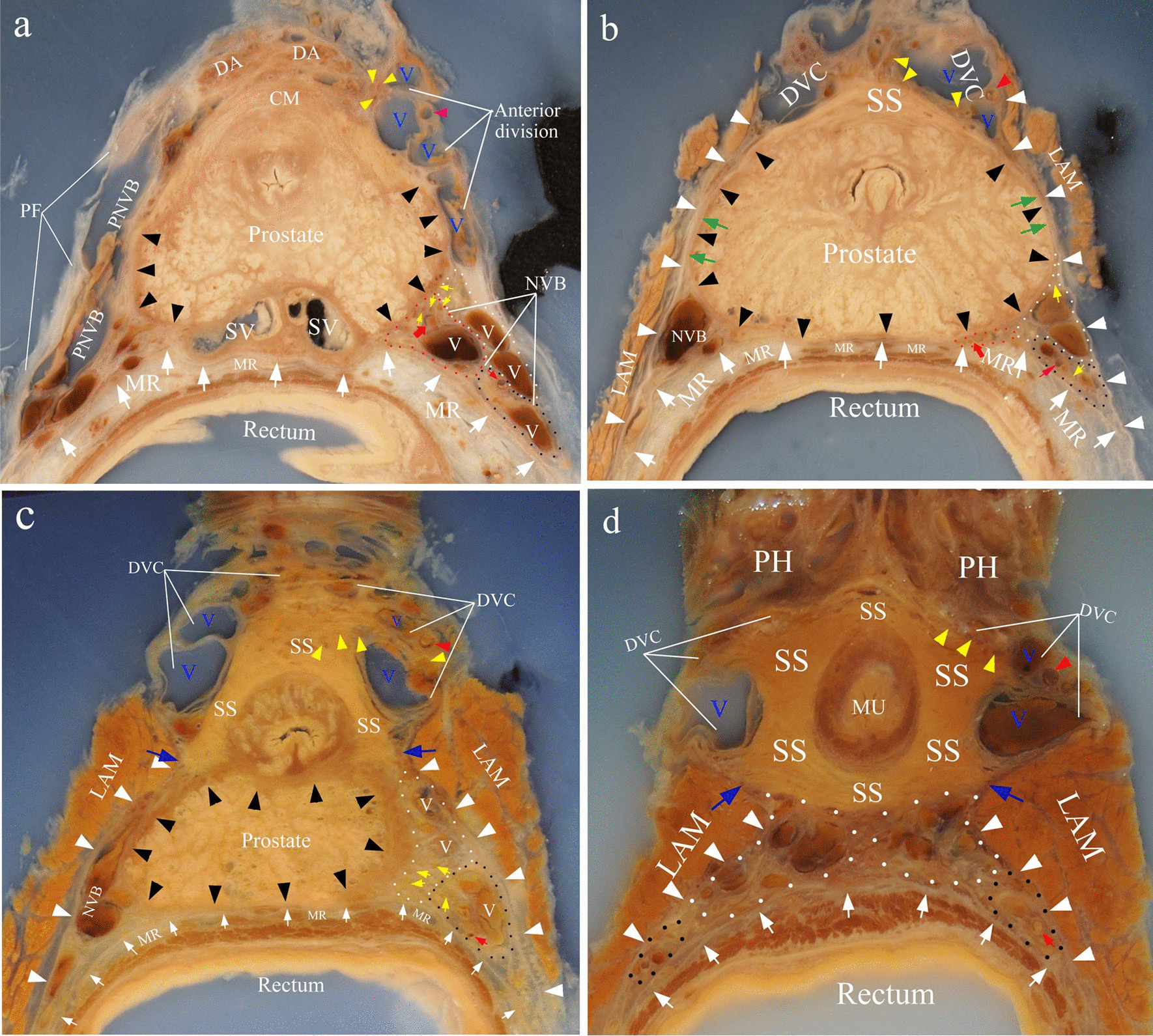
Axial celloidin sections. **a** The axial section through the bladder prostatic groove. **b** The section through the upper and middle prostate. Note that the capsule and the levator fascia adhered together at lateral aspect of prostate (green arrow). **c** The section through the prostatic apex. **d** The section through the membranous urethra. Blue arrow indicated the position where DVC (dorsal vascular complex) and CS (cavernous supply, circled by white dots) were separated by posterolateral portion of SS (striated sphincter). This portion of SS was attached to the outlet of LAM (Levator ani muscle). Black, white, red and yellow triangles indicated capsule, levator fascia, anterolateral branch of prostatic artery in DVC and nerves in DVC respectively; white arrows showed fascia proper of rectum; yellow arrows indicated nerves in NVB; broad red arrow indicated posterior-lateral branch of prostatic artery in NVB; red arrow indicated middle rectal artery; the prostatic supply was circled by red dots; the rectal supply was circled by black dots. PNVB, pelvic neurovascular bundle; PF, pelvic fascia; SV, seminal vesicles; MR, mesorectum; CM, circular muscle of detrusor; DA, detrusor apron; PH, penile hilum; V (blue), veins in DVC; V (white), veins in NVB

Axial sections through the bladder prostatic groove. The pelvic neurovascular bundle divided into anterior and posterior divisions (Fig. 1a). The posterior division was the NVB. The anterior division continued as the DVC which consisted of nerve fibers, dorsal veins and anterior-lateral pedicles of prostatic artery (Fig. 1a). NVB was composed of three supplies: the prostatic supply, the cavernous supply, and the rectal supply (Fig. 1a). The anterior surface of prostate was covered by circular muscle fibers (Fig. 1a). We have proved that these circular fibers originated from the detrusor [11]. The detrusor apron was located ventral to DVC.

Axial sections through the upper and middle prostate. Striated sphincter presented as a crescent shape and covered the anterior surface of prostate (Fig. 1b). Prostatic capsule covered the posterior and lateral surface of prostate. It was attached ventrally to the striated sphincter (Fig. 1b). The anterior portion of the levator fascia adhered laterally to the prostatic capsule. A fascia that surrounded the rectum and mesorectum was found (Fig. 1b). It was the fascia propria of rectum, which adhered tightly to the posterior surface of the capsule [12] (Fig. 1b). NVB was surrounded by a fascial triangle formed by levator fascia laterally, the fascia propria of rectum posteromedially and the prostatic capsule anteromedially (Fig. 1b). Prostatic supply went into prostate and became lesser, and only left the cavernous and rectal supply (Fig. 1b).

Axial sections through the prostatic apex and the membranous urethra. The prostatic capsule surrounded the dorsal, bilateral and ventral aspect of the prostatic stroma. The prostatic capsule and levator fascia were separated, and the cavernous supply went between them. The rectal supply ran posteroinferiorly between levator fascia and the fascia proper of rectum (Fig. 1c, d). Striate sphincter gradually embraced the urethra and finally completely surrounded the urethra (Fig. 1d).

**Fig. 2:**
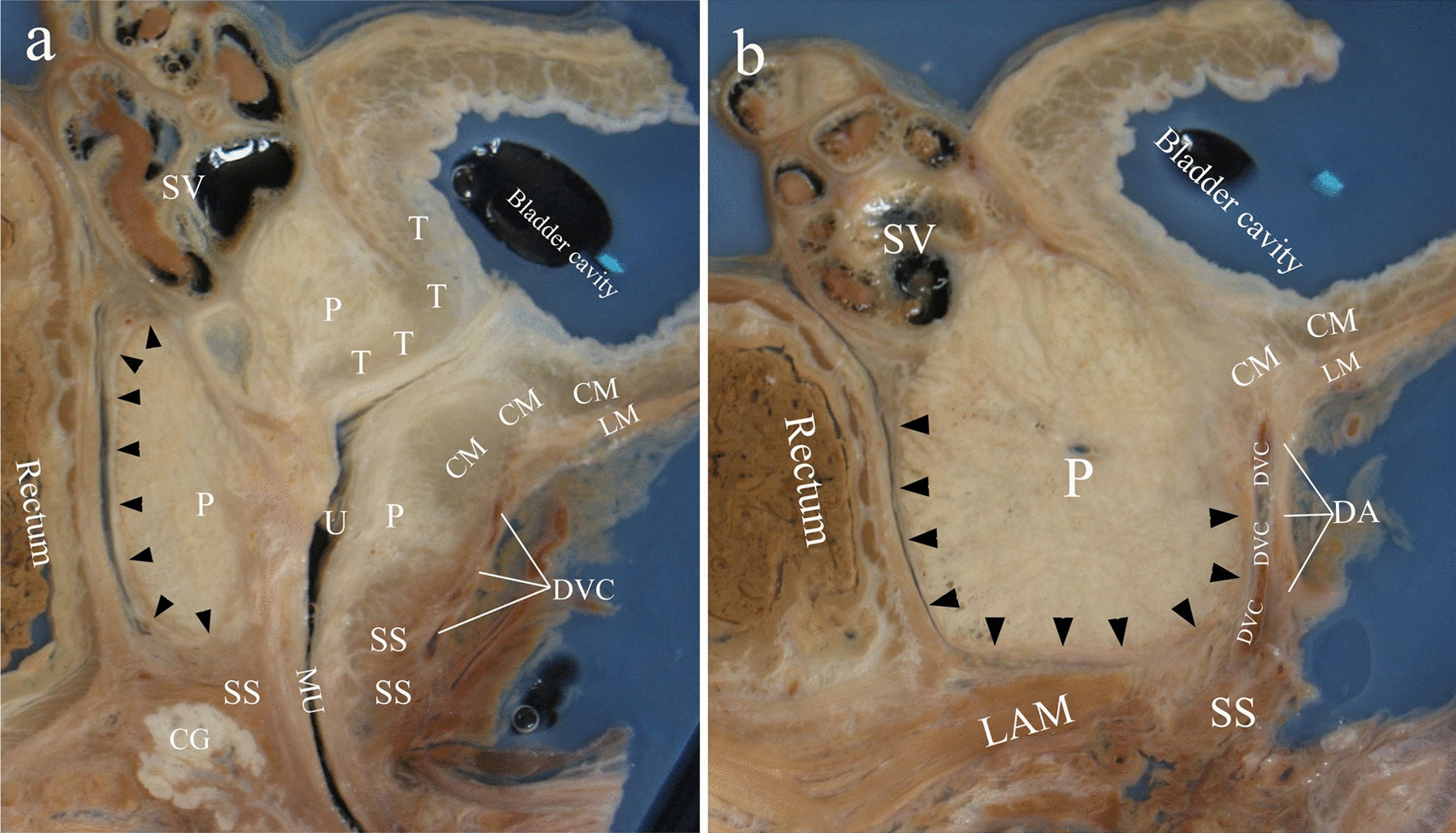
Saggital celloidin sections. **a** midsagittal section; **b** Section through lateral border of striated sphincter. Black triangles indicated the capsule. CM, circular muscle of detrusor; SS, striated sphincter; T, trigone muscle; LM, longitudinal muscle of detrusor; LAM, Levator ani muscle; DA, detrusor apron; DVC, dorsal vascular complex; P, prostate; SV, seminal vesicles; CG, Cowper’s glands; MU, membranous urethra

Sagittal sections of celloidin slices. On the midsagittal section, the dorsal surface of prostate was surrounded by the prostatic capsule. The capsule ended cranially at the root of seminal vesicles and caudally at the inferior surface of prostatic apex. The ventral surface of the prostate stroma was covered by circular muscle of detrusor and upper part of the striate sphincter muscle (Fig. 2a). On sections through lateral border of striate sphincter, the prostatic capsule surrounded the dorsal, inferior and ventral aspect of prostate. The detrusor apron originating from the longitudinal muscle of detrusor was attached to the pubic bone. DVC was located in the space between the prostatic capsule and detrusor apron (Fig. 2b).

**Fig. 3:**
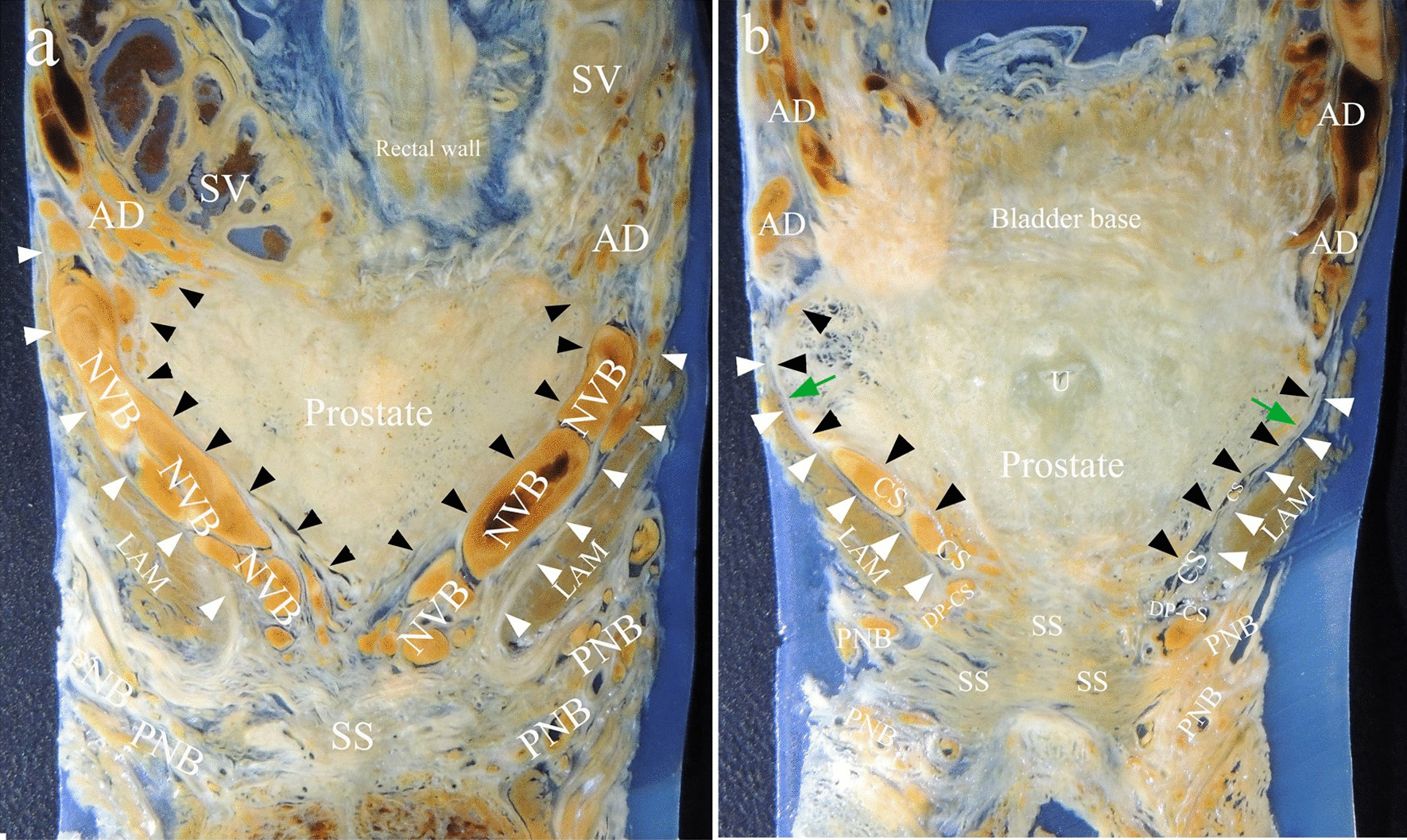
Coronal celloidin sections. **a** Section through seminal vesicles; **b** section through the posterior portion of striated sphincter. Note that the capsule and the levator fascia adhered together at the upper lateral aspect (green arrow). Black and white triangles indicated the capsule and the levator fascia respectively. SS, striated sphincter; AD, anterior division; CS, cavernous supply; DP-CS, the distal part of the cavernous supply; PNB, pudendal neurovascular bundle; SV, seminal vesicles; LAM, Levator ani muscle; U, urthra

Coronal sections of celloidin slices. On sections through the seminal vesicles, the prostatic capsule covered the bilateral and inferior surface of the prostate. NVB went between the capsule and the levator fascia (Fig. 3a). On sections through the posterior portion of striate sphincter, the capsule covered the lateral surface of prostate. It ended cranially at junction between the bladder and prostate, caudally at the junction between the prostate and striate sphincter. The anterior division ran in the groove between the bladder and prostate. The cavernous supply ran on the lateral aspect of lower prostate between the capsule and levator fascia. The distal part of the cavernous supply rounded the lower border of lavetor ani muscle and joined into the pudendal neurovascular bundle. The capsule and levator fascia adhered together at upper lateral aspect of the prostate (Fig. 3b).

**Fig. 4:**
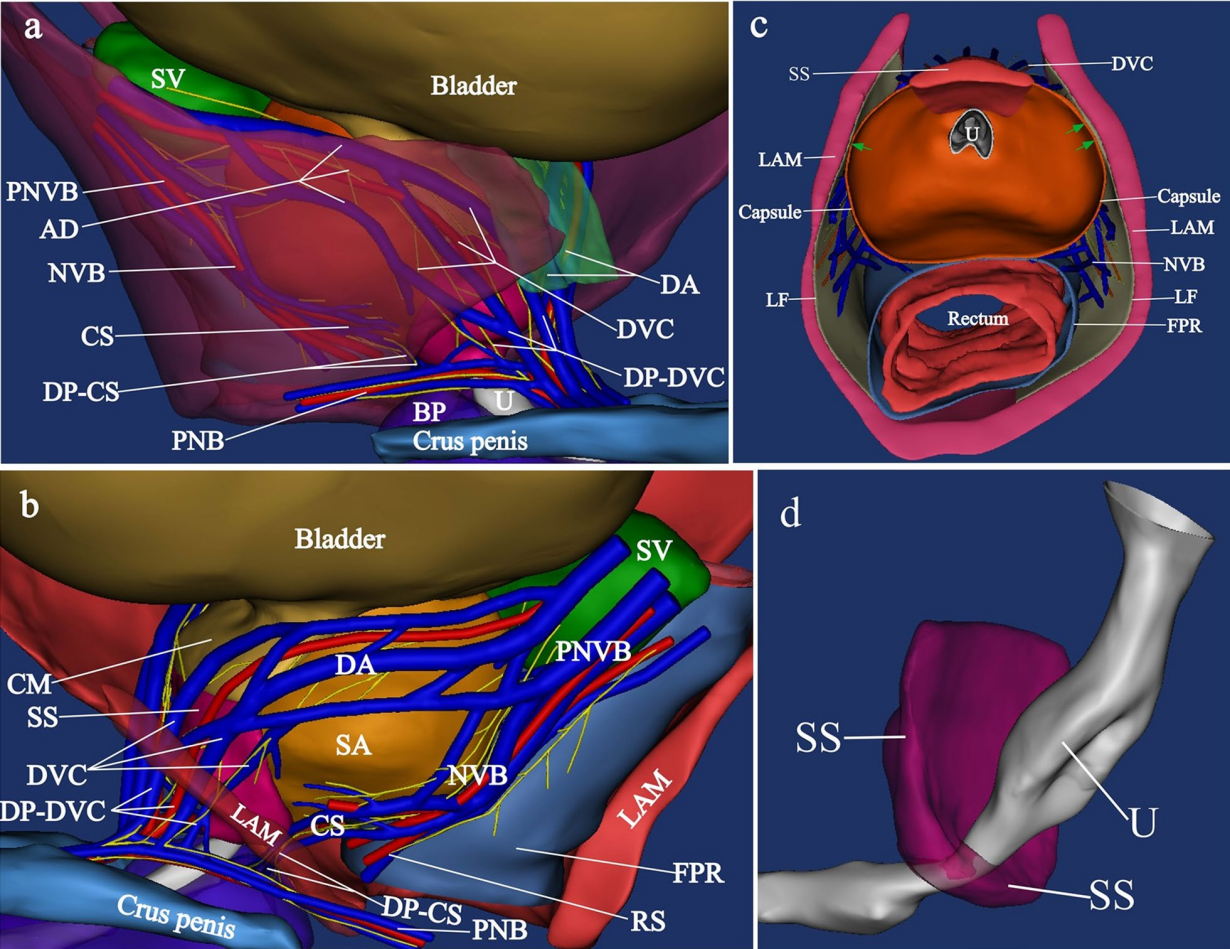
Three-dimensional reconstruction from transverse celloidin sections. **a** Lateral view showed the distal part of neurovascular bundles and their relationship with adjacent structure. LAM was translucent view. **b** Lateral view showed the neurovascular bundles lateral to prostate. The lateral part of LAM was removed. **c** A transversal section and superior view showed the pocket like capsule-AFMS complex. Note that the capsule and the levator fascia adhered together at the lateral aspect (green arrow). **d** Showed the lower part of striated sphincter completely embraced the urethral. PNVB, pelvic neurovascular bundle; PF, pelvic fascia; DVC, dorsal vascular complex; DP-DVC, the distal part of dorsal vascular complex; SV, seminal vesicles; MR, mesorectum; LAM, Levator ani muscle; CM, circular muscle of detrusor; DA, detrusor apron; SS, striate muscle; CS, the cavernous supply; DP-CS, the distal part of the cavernous supply; RS, the rectal supply; FPR, fascia proper of rectum; SA, safe area; BP, bulb of the penis; U, urethra; LF, levator fascia

3-Dimensional reconstruction of the structures. Pelvic neurovascular bundles were divided into the anterior and posterior divisions. The anterior division went anteriorly in the groove between bladder and prostate, and then ran inferiorly along the anterolateral surface of prostate to continuous as DVC. The distal part of DVC went anterolateral to the striate sphincter to enter the penile hilum. The posterior division was the NVB, which ran posterior and lateral to the prostate (Fig. 4a, b). NVB was split into the cavernous supply and the rectal supply. The cavernous supply went anteroinferiorly alongside the lateral surface of lower prostate. The distal part of cavernous supply ran between the posterolateral aspect of the striate muscle and anteromedial surface of levator ani muscle. It rounded the inferior border of levator ani muscle, and joined into the pudendal neurovascular bundle (Fig. 4a, b). The AFMS and the capsule together formed a pocket like structure accommodating prostate and urethra (Fig. 4c). The lower part of striated sphincter completely embraced the urethral (Fig. 4d).

## Discussion

Prostate stroma itself is immediately surrounded by a thin covering of tissue named as "capsule", which was absent at the anterior surface of prostate [2, 13]. Some researchers found that the "prostatic capsule" shifted to or showed a smooth transition to the anterior fibromuscular stroma [14, 15]. Our successive celloidin slices revealed that the bilateral ends of the capsule were attached to AFMS (Fig. 1a, b). 3-D reconstruction demostrated that the capsule and AFMS together formed a pocket like structure to accommodate the prostate and the urethra (Fig. 4c).

Recently, the prevailing view about the anatomy of the inferior part of the striate sphincter is that it is horseshoe-shaped or omega-shaped [16, 17]. Most researchers drew this conclusion from the serial slices of fetuses or children, and applied to adults [2, 18]. It was difficult to obtain successive sections of the pelvic organs of the adults. Although few authors tried to explore the striate sphincter on adults’ slice, they could not realize multiaxial and successive slices [19]. The successive horizontal celloidin slices showed that the complete closed striate sphincter encircled the membranous urethra (Fig. 1d). The sagittal and coronary slices revealed that the striate sphincter also existed dorsal to the membranous urethra (Fig. 2a, 3b). So, we conclude that the inferior part of the striate sphincter was ring shaped in male adults (Fig. 4d).

McNeal et al. defined a non-glandular tissue part of prostate as AFMS. It covered the anterior surface of the prostate and extended from the bladder neck to prostatic apex. But they did not confirm the origin of AFMS [20]. Brooks considered that AFMS migrated from the trigone [21]. Yucel and Baskin believed that the AFMS was a developmental component of the external sphincter [22]. Our team have proved that the upper part of AFMS originated from circular muscle of the detrusor [11]. The superior part of the striate sphincter covered the ventral surface of prostate and formed the lower part of the AFMS (Figs. 1b, c and 2a). Thus, the AFMS was composed of the circular muscle of the detrusor and the superior part of the striate sphincter (Fig. 4a). Thus, we believe that the AFMS is not a part of prostate.

Myers et al. proposed that the detrusor apron was a structure connecting the bladder to the pubis, and must be considered a major component of McNeal’s AFMS [23]. Our result displayed that the longitudinal muscle of detrusor extended downwards at the bladder neck to form the detrusor apron, and attached to the pubis (Fig. 2b). The upper part of AFMS originated from the circular muscle of detrusor, but the detrusor apron from the longitudinal muscle (Fig. 2). Additionally, there was a space between the detrusor apron and AFMS in which DVC was located (Figs. 1a, 2b and 4a). We presumed that the function of the detrusor apron was probably to keep the anteversion of bladder body. Preservation or reconstruction of the detrusor apron in radical prostatectomy probably contribute to the stability of bladder neck.

Villers et al. considered AFMS as a thickening of the capsule, and the two structures together circled the prostatic gland [24]. Observations of Elbadawi et al.’s confirmed the upper part of the striate sphincter as an integral component of the prostatic capsule. Muscular elements of the capsule probably contribute to the distal sphincteric mechanism of urethra [25]. Our results revealed that the capsule and AFMS formed a pocket like complex to accommodate the prostate and urethra (Fig. 4c). This complex may be a supplement of urinary control device at bladder neck and the membranous urethra. It is likely that the contraction of this complex results in sustained tension within the prostate, and then adds resistance to opening of the urethral conduit during filling to maintain continence.

More recent studies demonstrated variations of neural and vascular distribution lateral to the prostate. Walsh and Donker described a distinct NVB with bundle formation [1]. But some authors confirmed the dispersed neural distribution, such as spray-like or curtain-like [4, 26]. Tewari et al. proposed three zones of the periprostatic nerves: proximal neurovascular plate, the predominant NVB and accessory neural pathways [6]. Kiyoshima et al. found that the nerves and vessels existed with bundle or dispersion formations depending on the adhesion or separation of the lateral pelvic fascia and the prostatic capsule [14]. We found that vascular bundles lateral to prostate accompanying with nerve plexus and adipose tissue were located between the levator fascia and the capsule (Figs. 1, 3). But on the most convex region of the lateral prostate, the fascia and the capsule fused each other where we thought a safety area existed, because there was almost no adipose tissue and neurovascular bundles between them at this region (Figs. 1b, 3b). A careful capsule-levator fascia separation here may be more accordant with anatomical dissection in radical prostatectomy.

Al-Rifaei et al. reported the NVBs were divided in two parts near the prostate apex [27]. The anterior part crossed the forepart of the membranous urethra and entered the corpus cavernosum while the posterior part crossed the membranous urethra more posteriorly to enter the bulb of the penis. Alsaid et al. demonstrated that the anterior part was cavernous nerves, and the posterior part was corpus spongiosum nerves. They both originated from the spray-like nerve fibers on lateral prostate [28]. Our results revealed that the anterior part should be the distal part of DVC and the posterior part should be the distal part of CS (Fig. 4a, b). The distal part of DVC and the distal part of CS were separated by the posterolateral portion of striated sphincter (Fig. 4a, b). The distal part of CS was the communication between the pudendal neurovascular bundle and CS (Fig. 4a, b). Visceral nerve fibers in cavernous supply may join pudendal plexus via the distal part of CS to the corpus cavernosum. Somatic nerve fibers in pudendal nerve probably innervate the striated sphincter via the distal part of CS approach. When dealing with the apex of the prostate in nerve-sparing radical prostatectomy, we suggested to preserve the distal part of DVC and CS, which probably improved the postoperative continence and potency rates.

## Conclusions

The anterior neurovascular pathway from PNVB to the penile hilum included the anterior division and the DVC. The posterior neurovascular pathway was composed of the NVB, the cavernous supply and the pudendal neurovascular bundle (Fig. 4a, b). We presumed that simultaneous avoidance of the two pathways injury in nerve-sparing radical prostatectomy may maximally preserve postoperative sexual and urinary function.

## Data Availability

This study includes data and imaging from dead human bodies. For reasons of reverence, datasets used and/or analysed during the current study are available from the corresponding author on reasonable request.

## Abbreviations

AFMS: Anterior fibrous muscular stroma
CS: Cavernous supply
DVC: Dorsal vascular complex
NVB: Neurovascular bundle
PNB: Pudendal neurovascular bundle
PNVB: Pelvic neurovascular bundle
